# Outcomes of Cardio-Cerebral Infarction Patients: A Single-Centre Retrospective Study

**DOI:** 10.7759/cureus.72979

**Published:** 2024-11-04

**Authors:** Tamanna Agarwal, Julia Jenkins, Saranya Baleswaran, Mariana Nalmpanti, Aravinth Sivagnanaratnam

**Affiliations:** 1 Department of Stroke Medicine, Northwick Park Hospital, London North West University Healthcare NHS Trust, Harrow, GBR

**Keywords:** acute ischaemic stroke (ais), cardio-cerebral infarction, myocardial infarct, stroke, stroke management

## Abstract

Background

Cardio-cerebral infarction (CCI) belongs to an area of growing interest within cerebrovascular medicine as it refers to the concurrence of acute ischaemic stroke (AIS) and acute myocardial infarction (AMI) usually within 72 hours. A scenario where both the heart and brain sustain a catastrophic ischaemic insult can be devastating. We describe the incidence, treatment, and outcomes of CCI patients from a North London Hyper-Acute Stroke Unit (HASU) over two years and propose a potential treatment algorithm for the treatment of CCI.

Methodology

A retrospective analysis of AIS patients admitted to a North London HASU between January 2020 to December 2021 was performed to investigate the occurrence of concurrent AIS and AMI. Patients were initially included if they had elevated troponins upon admission to the stroke unit, following which they were excluded or included based on the diagnosis and treatment of myocardial infarction (MI) within 72 hours of AIS or vice versa. We describe the clinical characteristics of admission, clinical progression, management, and outcomes of patients suffering from CCIs.

Results

A total of 1,921 AIS patients were analysed. Of these, 302(16%) patients had elevated troponin and 35 (1.8%) patients were treated as acute coronary syndrome. Overall, 18 (0.9%) patients had CCI. Further analysis of CCI patients showed in-hospital death occurred in seven (41%) patients. The median length of stay was six days (range = 1-44 days). Angioplasty was used to treat MI in five (29%) patients, and the rest were medically managed or died.

Conclusions

Although rare, CCI has an exceedingly high mortality, and therefore, recognising and choosing the appropriate therapy is vital when attempting to re-perfuse both vital organs. Collaboration between stroke physicians, neuro-interventionalists, and cardiologists with a clear CCI pathway will enable better management and outcomes for these patients.

## Introduction

Stroke medicine, over the past few decades, has increasingly begun to describe the concurrence of acute ischaemic stroke (AIS) and acute myocardial infarction (AMI), a phenomenon referred to as cardio-cerebral infarction (CCI), a term originally coined by Omar et al. [1]. The diagnosis of either one of these medical emergencies carries a significant risk of adverse outcomes and the need for timely intervention. However, of even greater clinical concern, is a scenario where both the brain and heart sustain a catastrophic ischaemic insult, and decisions regarding the most appropriate therapy options when attempting to reperfuse both vital organs, without the expense of one over the other, will need to be made.

Time is a vital parameter, not only for the diagnosis or treatment of this life-threatening event but also for its categorisation. ‘Metachronous’ infarction describes one event preceding the other, and ‘synchronous’ infarction describes both events occurring at the same time [2]. ‘Hyper-acute simultaneous cardio-cerebral infarction’ refers to patients with simultaneous CCI, arriving within 4.5 hours of the thrombolytic therapeutic window [3]. Treatment of CCIs, thus, depends on their nature. The management of metachronous presentation has naturally focused on the preceding event, with subsequent treatment of the following insult, when it occurs [4].

Most strikingly, to date, there exist no definitive management guidelines for synchronous CCI. In these situations, there will always be a trade-off between rescuing the brain or heart first, and optimal treatment of one organ might lead to suboptimal treatment of the other or, in the worst case, cause further organ insult [5]. For example, on the one hand, the therapeutic dose for alteplase in the case of ST-elevated myocardial infarction (STEMI) is higher than that for AIS and requires a longer duration of administration, potentially increasing the risk of haemorrhagic transformation. On the other hand, the therapeutic dose of alteplase for AIS would be suboptimal for the treatment of non-ST-elevated myocardial infarction (NSTEMI) [3]. Due to the rarity of this presentation, and the absence of clinical trials providing clarity on evidence-based management options, the roadmap to revascularisation is a hazy one at best.

In this article, we aim to describe the incidence, treatment, and outcomes of synchronous and metachronous CCI identified in a North London Hyper-Acute Stroke Unit (HASU) over two years between 2020 and 2021. Additionally, we suggest a potential treatment algorithm for CCI patients in the hope of motivating further studies investigating the optimal management of these presentations.

This article was previously presented as a poster at the 10th European Stroke Organisation Conference on May 16, 2024.

## Materials and methods

Data collection and study design

This retrospective study was performed at the HASU at Northwick Park Hospital, which is a part of the London North West University Healthcare NHS Trust. Daily stroke admissions to the HASU were reviewed between January 2020 and December 2021, wherein clinical data for a total of 1,921 adult patients were analysed.

**Figure 1:**
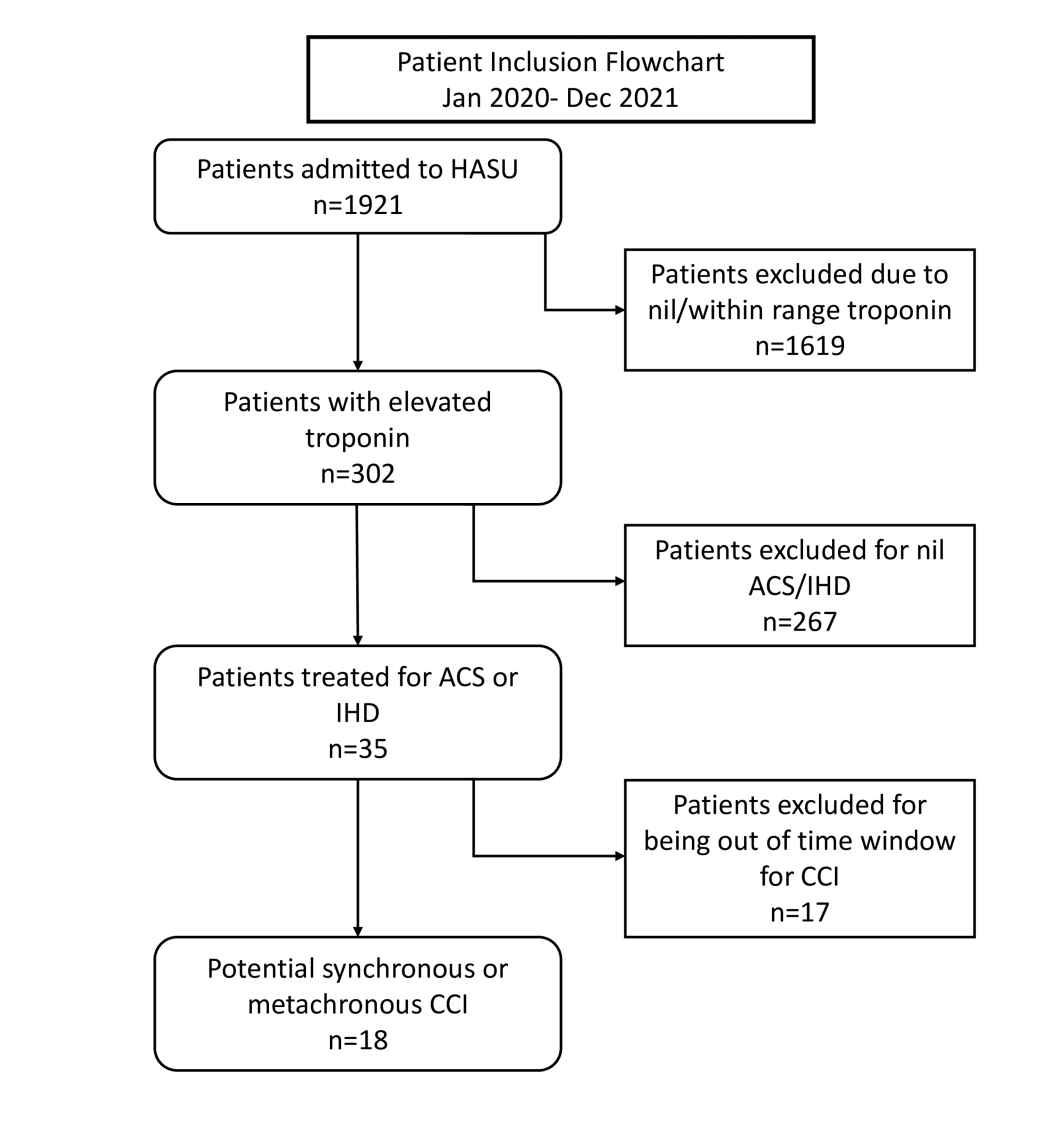
Patient inclusion flowchart. HASU = Hyper-Acute Stroke Unit; ACS = acute coronary syndrome; IHD = ischaemic heart disease; CCI = cardio-cerebral infarction

The patient inclusion flowchart can be seen in Figure 1. We initially included patients diagnosed with AIS, who had high troponin T (>15 ng/L) at admission or within 72 hours of stroke. Patients admitted or treated for haemorrhagic stroke were not included. Moreover, it is important to note that our unit does not regularly measure troponins, and it is only measured for clinical indications or to rule out cardiovascular involvement. AIS was diagnosed based on neurological symptoms and confirmed with relevant imaging, whereas the timing was determined based on the reported onset of symptomology or last seen well time. Myocardial infarction (MI) was diagnosed and timing was determined based on clinical presentation, cardiac enzyme levels, and/or evidence of MI on ECG or echocardiography. Patients were subsequently included or excluded based on their symptomology and treatment for acute coronary syndrome (ACS), ischaemic heart disease (IHD), or MI. Finally, patient inclusion was dependent on being within the time window for defined synchronous CCI.

Relevant data were extracted from the hospital medical records concerning demographic details, relevant medical history, cardiovascular risk factors (such as atrial fibrillation, smoking, hypertension, diabetes), prior anticoagulant use, and AIS and MI symptomology (STEMI or NSTEMI). Clinical progression and intervention were followed and categorised based on medical or interventional therapy, namely, intravenous thrombolysis, mechanical thrombectomy, and angioplasty. The study design was limited to in-patient death, and out-of-hospital mortality or death during a subsequent admission was not considered within the outcomes of this study.

The severity of stroke upon admission to our unit was scored between 0 and 42 according to the National Institutes of Health Stroke Scale (NIHSS), with a higher score indicating a greater severity of stroke. A score of less than 5 represents a minor stroke, a score between 5 and 15 represents a moderate stroke, a score between 16 and 20 represents a moderate-to-severe stroke, and a score between 21 and 42 represents a severe stroke. Baseline NIHSS scores, or those taken on admission, are considered to be strong predictors of clinical outcomes post-stroke [6,7].

As the data were collected as part of clinical care and anonymised for the purpose of this study, consent was not needed.

## Results

Data were analysed for a total of 1,921 patients admitted to the stroke unit over a period of two years. Of these, 302(16%) patients had elevated troponin and 35 (1.8%) patients were treated as ACS, IHD, or MI. Overall, 18 (0.9%) of these patients had CCI.

Patient demographic and clinical data can be seen in Table 1, and its comparison to the whole patient population is shown in Table 2. The median age of the CCI cohort was 82.5 years (range = 44-99 years) compared to the total patient population median age of 73 years (range = 19-105 years). The male-to-female ratio of the CCI cohort was predominately male (83% male, 17% female) compared to a more balanced ratio in the total patient population (56% male, 44% female). Suspected stroke aetiologies were largely cardioembolic with two anterior cerebral artery strokes, six middle cerebral artery strokes, six partial anterior circulation stroke syndrome/partial anterior circulation infarct strokes, five total anterior circulation strokes, and two lacunar stroke syndrome. The NIHSS was higher for the CCI cohort with a median NIHSS of 18 (range = 2-24), compared to the total population NIHSS of 5 (range = 0-42). Seven of the 18 patients suffered from a STEMI and the remaining 11 suffered from an NSTEMI during the incidence of CCI.

**Table 1:** Patient demographics and clinical characteristics. NIHSS = National Institutes of Health Stroke Scale; ACA = anterior cerebral artery; MCA = middle cerebral artery; OW = out-of-window; STEMI = ST-elevation myocardial infarction; PCI = percutaneous coronary intervention; RCA = right coronary artery; LBBB = left bundle branch block; NSTEMI = non-ST-elevation myocardial infarction; CKD = chronic kidney disease; T2DM = type 2 diabetes mellitus; HTN = hypertension; IHD = ischaemic heart disease; DAPT = dual antiplatelet therapy; PACS = partial anterior circulation stroke syndrome; TACI = total anterior circulation infarct; POCI = posterior circulation infarct; IP = in-patient; GORD = gastro-oesophageal reflux disease; LAD = left anterior descending artery; ICH = intracerebral haemorrhage; DVT = deep vein thrombosis; LACS = lacunar stroke

	Age	Gender	Comorbidities	Location of stroke	NIHSS (on admission)	Thrombolysis	MI	PCI or medical management	Length of stay (days)	In-Patient mortality
1	49	M	Missing	Left ACA infarct	4	No (OW)	STEMI	PCI: RCA stent (1 day)	16	No
2	99	M	Missing	Right MCA infarct	21	No (OW)	STEMI, new LBBB	Managed medically, too ill to intervene	4	No
3	81	M	CKD, T2DM, HTN, IHD	Left PACS	4	No (OW)	NSTEMI	PCI and DAPT (11 days)	20	No
4	89	M	Missing	Left MCA infarct	21	No (on anticoagulation)	NSTEMI	Managed medically, too ill to intervene	1	Yes
5	89	F	HTN, T2DM, dementia	Left TACI	20	No (OW)	NSTEMI	Managed medically (significant comorbidities)	18	No
6	69	M	HTN	POCI	2	No	NSTEMI	Managed medically, refused IP angio	6	No
7	78	M	T2DM, HTN, IHD	Right PACI	4	No	NSTEMI	PCI and DAPT	6	No
8	77	F	IHD, T2DM	Left MCA, ACA, and cerebellar infarct	21	No	NSTEMI	Medically managed	2	No
9	92	M	Missing	Left MCA infarct/TACI	24	No	NSTEMI	Medically managed	6	Yes
10	90	F	Missing	Left PACI	21	Yes	STEMI	PCI: LAD, RCA stenting (2 days), and DAPT	14	No
11	44	M	T2DM, GORD	Right MCA infarct, TACS	18	No (contraindicated due to extent of stroke- tirofiban infusion for 3 days for AIS)	Anterior STEMI	PCI: LAD (3 days)	44	No
12	77	M	Pancreatic CA, DVT	Subacute infarct, right occipital lobe, PACS	3	No (OW)	STEMI	Medically managed	4	No
13	82	M	IHD	Right LACS	9	No (OW)	STEMI	PCI: 4x stents to LAD	9	No
14	53	M	None	Lacunar infarct + left thalamic ICH + R ICH	4	Yes	NSTEMI	Medically managed	1	Yes
15	90	M	Prostate CA, CKD,	Left LACS	19	No (OW)	NSTEMI	?	3	Yes
16	86	M	HTN, T2DM, stroke	Right TACS	19	No	NSTEMI	Medically managed	7	Yes
17	83	M	HTN, Dementia	TACS	18	No	STEMI	Too ill to intervene	4	Yes
18	85	M	HTN, dementia	Right MCA infarct (R2 thrombus), PACS	9	No (OW)	NSTEMI	Medically managed	6	Yes

**Table 2:** Comparison of cohort and population demographics. NIHSS = National Institutes of Health Stroke Scale; CCI = cardio-cerebral infarction

Clinical characteristic	CCI cohort	Total population
Median age in years	82.5	73
Age range in years	44–99	19–105
Male patients	15 (83%)	1,076 (56%)
Female patients	3 (17%)	845 (44%)
Median NIHSS	18	5
NIHSS range	2–24	0–42
Patients who underwent thrombolysis	2 (11.1%)	323 (16.8%)
Patients who did not undergo thrombolysis	16 (88.9%)	1,598 (83.2%)

Analysis of employed interventions revealed that angioplasty was used to treat MI in six (33%), patients and the rest were medically managed or died. Only two (11.1%) patients were thrombolysed, while others were not due to being out of the thrombolysis window or not meeting the other conditions for treatment. This is compared to a 16.8% thrombolysis rate in the total population. Additionally, in-hospital death occurred in seven (38.9%) patients, and the median length of stay was six days (range = 1-44 days).

## Discussion

In this retrospective analysis of AIS patients, we investigated the possibility of synchronous and metachronous CCI. Our study adds to the small pool of available data on CCI. We found only a very small fraction of patients over two years with CCI out of a large cohort of AIS patients admitted to our unit. The treatment employed varied on a case-by-case basis, representing the complexity of standardising CCI treatment when considering patient-specific factors, severity of insult, and timing of injury (out-of-hospital or not). Outcomes also ranged widely, while some patients made a near-complete recovery, others suffered in-hospital deaths. Despite low incidence, our CCI patients’ data indicate a mortality rate of 38.9% over two years compared to an annual average of 12% in patients with stroke alone (similar to the national average). The high mortality rate can be attributed not only to the high mortality and morbidity of CCI, as it is a severe medical condition, but also to the older median age and higher NIHSS on presentation of these patients compared to patients admitted to our unit only for stroke. As CCIs present a rare clinical dilemma, meta-analyses of this condition are far and few. However, a recent meta-analysis conducted by Ng et al. reported similar levels of mortality as well as a male predominance of patients in the studies analysed [8].

Our study confirms existing literature reporting a low incidence of CCI. A three-year prospective study on AIS patients admitted to a geriatric unit showed that 12.7% were found to have AMI within 72 hours of admission [9]. And vice versa, the Global Registry of Acute Coronary Event (GRACE) trial reported an incidence of in-hospital stroke as 0.9% for patients admitted with ACS [10]. Despite statistics for metachronous CCI being available, there exists a paucity of data on the incidence of synchronous CCI, with only a study by Yeo et al. describing synchronous CCI [11]. Yeo et al. also reported a proportionate occurrence of CCI patients within an AIS patient cohort, recording five cases out of a total of 555 AIS patients, compared to our identification of 18 CCI cases among 1,921 AIS patients.

CCI has been reported anecdotally, as well as within case series; however, its pathophysiology remains unclear. One author cites it as a possible complication of COVID-19, while another reports it to be a complication of domestic low-voltage electrical injury [5,12,13]. Several mechanisms have been proposed to play a role in the occurrence and pathophysiology of CCI. In the context of left ventricular systolic dysfunction or hypokinesia of the ventricular myocardium, subsequent development of mural thrombus may occur, which, in turn, is at risk for embolisation to both coronary and cerebral arteries [11]. Similarly, in atrial fibrillation, a thrombus may also embolise to vascular territories [14]. In patients with a patent foramen ovale, a deep vein thrombosis or right ventricular thrombus may also cause CCI in this fashion [1]. Furthermore, circulatory collapse in the context of AMI, in a patient with longstanding hypertension and cerebral auto-regulation, may result in a reduction in cerebral blood flow resulting in a watershed infarct. An ascending or type-1 aortic dissection, with the dissection flap extending to both coronary and carotid arteries, has been described as the cause of AMI and stroke [15].

Finally, another proposed mechanism of CCI relates to brain-heart dysregulation. The insular cortex has been shown to play a critical role in central autonomic system regulation. Because the insular cortex is closely related to the middle cerebral artery, its structure is exposed to a higher rate of cerebrovascular disease. Damage to the insular cortex may cause arrhythmia, myocardial injury, and higher plasma levels of catecholamine [16]. Cardiac sympathetic overactivity may induce diffuse myocardial injury (myocytolysis) [4], or in the context of AIS with adrenergic surge, possibly even stress-induced myocardial stunning (Takosubo syndrome) which would favour ventricular thrombus formation [3]. One study by Kim et al. attempted to differentiate between the causative factors of synchronous and metachronous CCIs, suggesting metachronous CCIs have diverse underlying mechanisms whereas a synchronous CCI is secondary to an embolism from a preceding MI [17].

As our findings suggest, the shortcomings of therapeutic options in the context of AIS and AMI relate largely to issues of timing, dosage, and duration of thrombolysis. In addition, there are no evidence-based guidelines available detailing the correct management of CCI. Despite thrombolysis being the common therapeutic option for AIS and AMI, the timing of administration for AIS would necessitate administration within 4.5 hours since the onset of acute neurological fallout, and the indication for thrombolysis in AMI is only relevant for STEMI, with higher rates of cardiac wall rupture in NSTEMI. Furthermore, the dose of alteplase for STEMI is higher than in AIS and is administered for a longer duration, creating a higher risk of haemorrhagic transformation in AIS. However, currently, data confirming this increased risk is lacking [5].

The treatment of AIS based on the American Heart Association/American Stroke Association (AHA/ASA) guidelines for the early management of patients with AIS (2019) primarily recommends the use of intravenous (IV) alteplase for thrombolytic therapy administered as 0.9 mg/kg, with a maximum dose of 90 mg over 60 minutes following an initial 10% bolus within the first minute [18]. This applies not only to patients presenting within three hours of symptom onset or last known well but also to patients falling under the 3-4.5 hours window. Mechanical thrombectomy is recommended for patients presenting between 0-6 hours if they meet the criteria outlined within the guidelines (including NIHSS score of >/=6, prestrike modified Rankin Scale (mRS) score of 0-1, causative occlusion of the internal carotid artery or middle cerebral artery segment). Stent retriever and direct aspiration forms of thrombectomy are described as non-inferior to each other. Further, eligible patients are recommended to undergo mechanical thrombectomy within 6-24 hours of symptom onset.

The treatment of STEMI and NSTEMI is based on the European Society of Cardiology guidelines for the management of ACS [19]. STEMI therapy centres reperfusion therapy for all patients with a working diagnosis of STEMI and symptoms of ischaemia within 12 hours of onset. A percutaneous coronary intervention (PCI) is recommended as first-line therapy if the time from diagnosis to PCI is less than 120 minutes. PCI is further recommended for NSTEMI patients with a high index of suspicion for unstable angina.

A combined statement from the AHA/ASA recommended a dose of 0.9 mg/kg of IV alteplase (maximum 90 mg and 10% of the dose administered as a bolus, and the remainder as an infusion over 60 minutes) for both presentations, when acute stroke symptom onset was within three hours, followed by primary coronary angioplasty (PCA) [2,20]. PCA is considered the first-line therapy for STEMI, and some cases of NSTEMI. However, it must be kept in mind that PCI before thrombectomy may reduce good neurological recovery, while delaying PCI may increase irreversible myocardial damage. Of further note is the high doses of antiplatelets and anticoagulation co-administered with PCI as a part of standard therapy, which can also increase the risk of haemorrhagic transformation within these patients [4].

Endovascular treatment of AIS in selected patients, following IV thrombolytic therapy, has also been associated with significantly improved functional independence compared with IV treatment alone [21]. Therefore, a combined endovascular approach for both AIS and AMI with visualisation of both coronary and cerebral arterial occlusions (with or without IV thrombolysis depending on the time since onset of symptoms) has been suggested and may be an effective therapeutic solution after considering the specific need for each organ [3,11].

**Figure 2:**
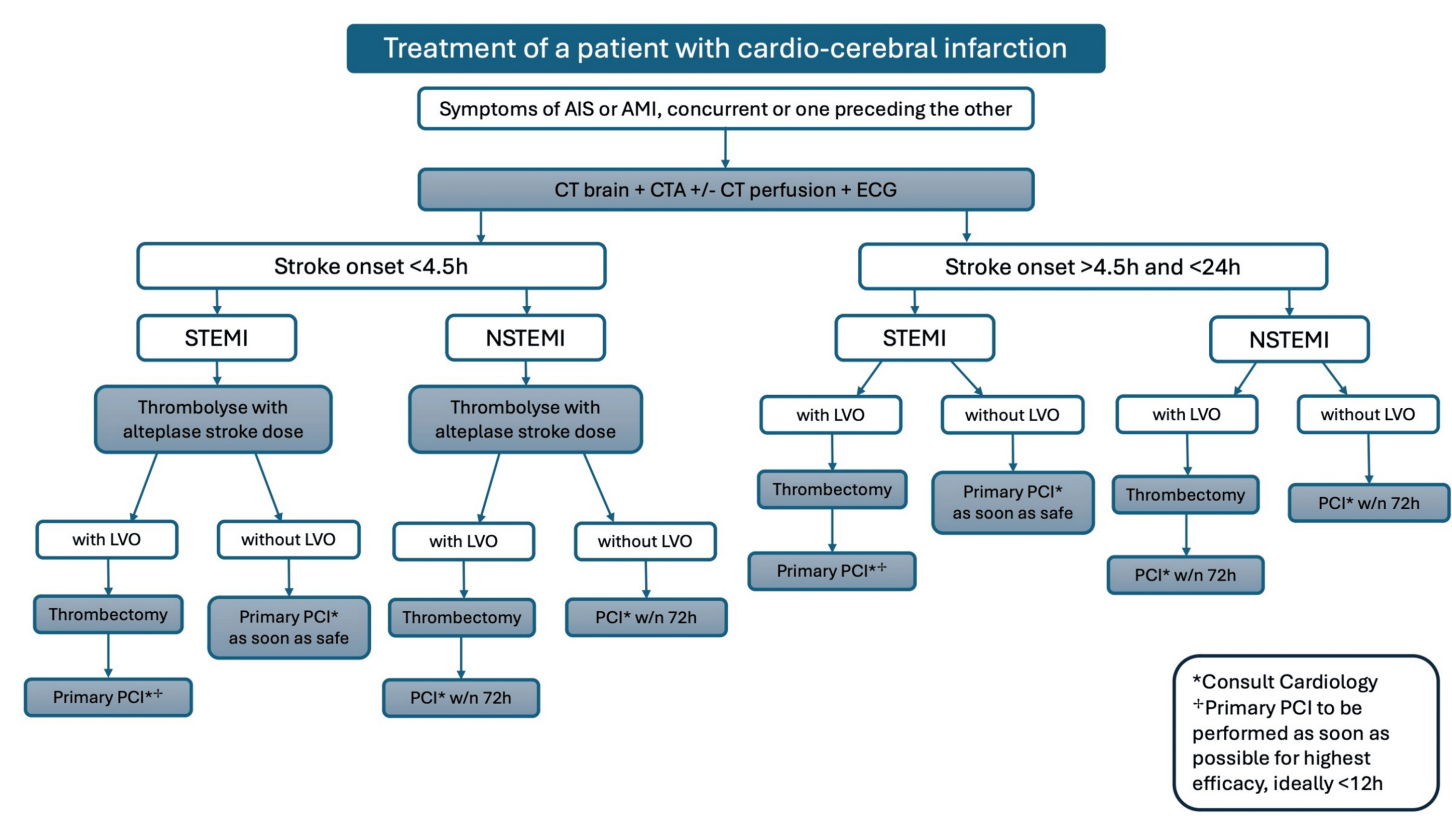
Proposed treatment algorithm for a patient with cardio-cerebral infarction. AIS = acute ischaemic stroke; AMI = acute myocardial infarction; CTA = CT angiogram of carotids, vertebrals, and intracranial vessels; STEMI = ST-elevation myocardial infarction; NSTEMI = non-ST-elevation myocardial infarction; LVO = large-vessel occlusion; PCI = percutaneous coronary intervention Image credits: Tamanna Agarwal.

Being a high-mortality illness with no evidence-based guidelines to treat the various subtypes of AIS and AMI led us to create a treatment algorithm with the help of a cardiologist colleague. We used relevant existing guidelines for AIS and AMI where necessary and proposed the following treatment algorithm presented in Figure 2, considering the window of presentation and management possibilities based on the nature of stroke (large-vessel occlusion or not) and nature of MI (STEMI or NSTEMI). Time being the major limiting factor in questions of reperfusion with tissue plasminogen activator, it is crucial that these patients are managed using the appropriate intervention as quickly as possible to minimise tissue loss and maximise chances of recovery and reduce morbidity. Being a condition requiring a multidisciplinary approach, it is also essential to consult and discuss with the cardiology team the approach to the patient’s MI and its management.

In a case study by Plata-Corona et al., successful revascularisation was performed on a patient with non-hyperacute CCI, with initial low-dose intra-arterial thrombolysis (due to presentation outside of the AIS thrombolysis window), with subsequent mechanic thrombectomy and coronary angiography with placement of a drug-eluting stent. The patient in this study made a full recovery, with an mRS score of 0 and a normal transthoracic echocardiogram at 30 days post-discharge [2]. The outcome of this study highlights the importance of individualised therapeutic options, within the context of CCI.

Our study is subject to a few limitations. Firstly, being a single-centre study with a small CCI sample size, the generalisability of our findings may be limited. Second, there were instances where clinical data for patients were incomplete. Furthermore, the rare occurrences of silent MI could potentially not be caught clinically as troponins were assessed for clinical indications, and as troponins were not measured for every admitted stroke patient, our data may also represent an underdetection of concurrent AMI with stroke. Lastly, out-of-hospital MI/AIS are hard to strictly categorise as metachronous or synchronous CCIs.

## Conclusions

CCIs, though occurring rarely, have a high morbidity and mortality rate and require rapid intervention. It is critical to choose the appropriate therapy that reperfuses and protects both the heart and brain from further ischaemic insult and damage. Multidisciplinary collaboration between the involved fields of cerebrovascular medicine, namely, stroke, cardiology, and interventional neuroradiology, should shed light on effective therapy modalities and thus improve outcomes for these patients.
